# Pteryxin enhances human NK-cell cytotoxicity by upregulating NKp30, NKp46, and 2B4 via ERK/AKT signaling

**DOI:** 10.3389/fphar.2025.1698826

**Published:** 2026-01-21

**Authors:** Eun Sun Park, Jong-Tae Kim, Yo-Sep Hwang, Jahyeong Han, Hyo-Min Park, Hyang Ran Yoon, Hee Jun Cho, Hee Gu Lee

**Affiliations:** 1 Immunotherapy Research Center, Korea Research Institute of Bioscience and Biotechnology (KRIBB), Daejeon, Republic of Korea; 2 Department of Biomolecular Science, KRIBB School of Bioscience, Korea University of Science and Technology (UST), Daejeon, Republic of Korea

**Keywords:** cancer, ERK/AKT, immunotherapy, natural killer cells, pteryxin

## Abstract

Natural killer (NK) cells play critical roles as effector cells by directly identifying and killing virus-infected and cancer cells. Pteryxin exhibits diverse antioxidant and anti-inflammatory effects; despite its known properties, the influence of pteryxin on NK cells is not understood fully. In this study, we evaluated the potential of pteryxin to enhance the cytotoxicity of NK cells. Pteryxin markedly enhanced the cytotoxic activities of both NK-92 and primary human NK cells against leukemia and colorectal cancer cell lines in a dose-dependent manner. Furthermore, it elevated the surface expression of key activating receptors NKp30, NKp46, and 2B4 in the NK-92 cells. This upregulation was accompanied by activation of the ERK and AKT signaling pathways, leading to increased production of cytotoxic mediators, including granzyme B and perforin. Moreover, *in vivo* studies using the CT26 mouse model revealed that pteryxin administration inhibited tumor growth in a dose-dependent manner. NK cells from the pteryxin-treated mice demonstrated enhanced cytotoxicity against YAC-1 leukemia cells. The anticancer effects of pteryxin were abolished when the NK cells were significantly reduced using anti-asGM1 antibody, confirming the critical role of the NK cells in its antitumor activity. Collectively, these findings demonstrate that pteryxin stimulates the ERK and AKT signaling pathways to enhance NK cell cytotoxicity against tumors, supporting its potential as a novel enhancer of NK-cell-driven antitumor responses.

## 1 Introduction

Natural killer (NK) cells are innate lymphocytes that exert potent cytotoxic activities against virus-infected and transformed cells in an antigen-independent manner (Fang et al., 2017). They account for approximately 5%–15% of the peripheral blood lymphocytes and are enriched in tissues like the bone marrow, spleen, liver, lungs, uterus, and secondary lymphoid structures (Freud et al., 2017). Unlike T lymphocytes, which require antigen recognition via the major histocompatibility complex (MHC) molecules for activation, NK cells coordinate their cytotoxic responses by integrating signals from a diverse repertoire of activating and inhibitory receptors (Pegram et al., 2011). NK cells exhibit cytotoxicity when stimulated by the absence of the autologous MHC I on tumor cells (Debska-Zielkowska et al., 2021; Wang et al., 2021). NK cells express inhibitory killer immunoglobulin-like receptors (KIRs) that recognize the MHC I molecules commonly found on the surfaces of normal cells. The presence of MHC I generates inhibitory signals that protect healthy cells from NK-cell-mediated attacks. Conversely, activation is facilitated through receptors, such as activating the KIRs, NKG2D, natural cytotoxicity receptors (NCRs), DNAM-1, and 2B4, which recognize stress-induced ligands on the aberrant cells (Chan et al., 2014; Vivier et al., 2011). The balance between these opposing receptor-mediated signals is critical in determining the functional state of the NK cells and represents a tightly regulated mechanism by which the NK cells discriminate between healthy and diseased cells (Pegram et al., 2011).

Upon activation, the NK cells employ multiple cytotoxic mechanisms to eliminate aberrant target cells. One of the primary modes of action here involves granule-mediated cytotoxicity, where perforin facilitates transient pore formation in the target cell membrane to enable the entry of granzymes and granulysin that subsequently trigger apoptotic pathways (Grudzien and Rapak, 2018). In addition to granule exocytosis, the NK cells can trigger apoptosis via engagement of death receptors like the Fas ligand (Fas L) and tumor-necrosis-factor-related apoptosis-inducing ligand (TRAIL) (Ramirez-Labrada et al., 2022). The NK cells also mediate antibody-dependent cellular cytotoxicity (ADCC) through CD16 (FcγRIIIa), which recognizes the antibody-coated targets and transduces activating signals to ensure target cell lysis (Wang et al., 2021). Beyond direct cytotoxicity, the NK cells play pivotal immunomodulatory roles within the tumor microenvironment (Shimasaki et al., 2020). They act as a functional bridge between innate and adaptive immunity by secreting a broad spectrum of cytokines and chemokines that activate and recruit other immune cell populations (Hamilton and Plangger, 2021; Smyth et al., 2002). For example, NK-cell-derived interferon-γ (IFN-γ) promotes T helper 1 (TH1) polarization and enhances the cytolytic potential of CD8^+^ T cells (Martin-Fontecha et al., 2004). Furthermore, apoptotic tumor cells targeted by the NK cells can serve as antigen sources for dendritic cells (DCs), promoting their maturation and subsequent presentation of tumor antigens to the T cells, thereby initiating robust adaptive immune responses (Nguyen-Pham et al., 2012).

Given the central roles of the NK cells in tumor surveillance and immune regulation, multiple therapeutic strategies have been devised to potentiate NK-cell-mediated cytotoxicity by either enhancing the activating pathways or disrupting the inhibitory signals (Barnes et al., 2021). Current NK-based immunotherapies include chimeric-antigen-receptor-engineered NK (CAR-NK) cells, NK-cell engagers, immune checkpoint blockade, and small-molecule approaches to improve NK cell expansion and functions (Laskowski et al., 2022; Biederstadt and Rezvani, 2025; Jing et al., 2025). Among these strategies, therapies based on natural compounds have gained attention owing to their ability to modulate immune signaling. Several phytochemicals, including curcumin, metformin, and resveratrol, have been reported to enhance NK-cell cytotoxicity, primarily through the classical inflammatory signaling pathways like JAK/STAT or p38/NF-κB. For example, curcumin has been shown to enhance NK-cell cytotoxicity against MDA-MB-231 human breast carcinoma cells by activating the STAT4 and STAT5 signaling pathways (Lee and Cho, 2018); similarly, metformin has been found to promote NK-cell activation via the induction of p38 mitogen-activated protein kinase (MAPK) signaling (Xia et al., 2021).

Pteryxin is a coumarin-derived natural compound extracted from the roots of *Peucedanum praeruptorum* that has been reported to exhibit neuroprotective, antiobesity, and antioxidant properties (Nugara et al., 2014; Patel and Patel, 2024). Recent studies have demonstrated that pteryxin effectively attenuates lipopolysaccharide (LPS)-induced inflammatory responses in RAW 264.7 macrophages and significantly ameliorates LPS-induced acute lung injury (ALI) *in vivo* (Zhen et al., 2022; Xuan et al., 2022). These findings suggest that pteryxin modulates immune responses through MAPK-related signaling, which differs from previously studied NK-enhancing phytochemicals that act primarily via the STAT or NF-κB pathways. However, it remains poorly defined whether pteryxin directly affects NK-cell activation or cytotoxic functions. In the present study, we provide evidence that pteryxin markedly enhances NK-cell-mediated cytotoxicity. Mechanistically, pteryxin treatment was associated with activation of the MAPK signaling cascade. Furthermore, pteryxin potentiated NK cell effector functions through the perforin- and granzyme-dependent pathways, along with increased expression of the activating receptor NKp30. The present study demonstrates a novel immunoregulatory role of pteryxin and provides evidence supporting its application in augmenting NK-cell-based cancer immunotherapy.

## 2 Materials and methods

### 2.1 Cells

All cell lines used in the study were acquired from American Type Culture Collection (ATCC). The NK-92 cell line was cultured in MEM-a supplemented with 12.5% heat-inactivated fetal bovine serum (FBS; Gibco), 12.5% heat-inactivated horse serum (Gibco), 0.2 mM of myo-inositol (Sigma-Aldrich), 0.1 mM of 2-mercaptoethanol (Sigma-Aldrich), 0.02 mM of folic acid (Sigma-Aldrich), 1% antibiotics, and 20 ng/mL of interleukin 2 (IL-2). For the pteryxin treatment, the NK-92 cells were incubated with various doses of pteryxin along with 10 ng/mL of IL-2. The K562 and YAC-1 cell lines were maintained with RPMI medium containing 10% heat-inactivated FBS and 1% antibiotics, while the SW620, SW480, HT-29, and KMRC cell lines were cultured in DMEM with 10% heat-inactivated FBS and 1% antibiotics.

Human peripheral blood NK cells were obtained from STEMCELL Technologies. The primary NK cells were expanded using the ImmunoCult NK Cell Expansion kit (STEMCELL Technologies) according to manufacturer protocols and sustained in RPMI medium supplemented with 10% heat-inactivated FBS and 40 ng/mL of IL-2 (PeproTech).

All cells were cultured at 37 °C in a humidified atmosphere containing 5% CO_2_.

### 2.2 Cell counting kit (CCK)-8 cell viability test

The cells were seeded onto 96-well plates and exposed to different concentrations of pteryxin for either 24 h or 48 h. Subsequently, approximately 20 µL of CCK-8 reagent (Dojindo Molecular Technologies) was added to each well; after incubating for 4 h at 37 °C, the absorbance was measured at 450 nm using a microplate reader.

### 2.3 Cytotoxicity assay

The CytoTox 96 Non-Radiative cytotoxicity assay (Promega, Madison, WI, United States) was used to assess lactate dehydrogenase (LDH) activity. Here, the NK-92 (effector) cells were first treated with pteryxin for 48 h, and the target cells (5 × 10^3^ cells/well) were co-incubated with drug-pretreated effector cells at different ratios. Following incubation, the supernatant (50 µL) was collected from each well, and the CytoTox 96 reagent was added and incubated for 30 min in the dark. The enzymatic reaction was stopped using a stopping solution (50 µL), and the absorbance was measured at 490 nm. The results were then expressed as percentage of cytotoxicity calculated using the below formula:

$$\frac{\left(\text{experimental}-\text{spontaneous}\;\text{release}\;\text{of}\;\text{effector}\;\text{cells}-\text{spontaneous}\;\text{release}\;\text{of}\;\text{target}\;\text{cells}\right)}{\left(\text{maximum}\;\text{release}-\text{spontaneous}\;\text{release}\right)}\times 100.$$

The cytotoxicity was determined by the calcein-AM release assay. In brief, the K562 target cells were stained with calcein-AM for 1 h at 37 °C; pretreated NK-92 and primary NK cells were used as the effector cells and co-cultured with calcein-AM-stained K562 cells at various ratios. After incubation, the absorbance values were measured at excitation and emission wavelengths of 485 nm and 538 nm, respectively, using a microplate reader. The results were then expressed as percentage of cytotoxicity calculated using the following expression:

$$\frac{\left(\text{experimental}\;\text{release}-\text{minimum}\;\text{release}\right)}{\left(\text{maximum}\;\text{release}-\text{minimum}\;\text{release}\right)}\times 100.$$

### 2.4 Polymerase chain reaction (PCR)

The total RNA was extracted using a total RNA extraction kit (Biofact), and oligo-dT (Bioneer) was used as a primer for cDNA synthesis. Then, quantitative PCR was conducted with the Accupower 2x Greenstar qPCR MasterMix (Bioneer) and analyzed by StepOnePlus real-time PCR (RT-PCR; Thermo Fisher Scientific). The primers used in this step are listed in Table 1, and the relative expression levels of the target genes were determined using the 2^−ΔΔCT^ method. All samples were normalized to the housekeeping gene β-actin.

**TABLE 1:** List of primers used for quantitative real-time polymerase chain reaction (qRT-PCR) in this study.

Gene name	Forward	Reverse
β-Actin	CAA​ACA​TGA​TCT​GGG​TCA​TCT​TCT​C	GCT​CGT​CGT​TCG​ACA​ACG​GCT
NKp46	AGA​ATC​TCC​TTC​GGA​TGG​GC	GGT​CCA​ACA​CAG​AGC​TCA​CG
NKp44	TAC​CCA​AAA​AGC​CAC​CTG​CC	GTG​TGT​TCA​TCA​TCA​TCA​TCG​CT
NKp30	TTT​CCT​CCA​TGA​CCA​CCA​GG	GGA​CCT​TTC​CAG​GTC​AGA​CAT​T
NKG2D	GTT​ACT​GTG​GCC​CAT​GTC​CT	AGA​AGG​CTG​GCA​TTT​TGA​GA
2B4	TCT​ACT​GCC​TGG​AGG​TCA​CCA​G	GAC​CAA​GCA​AGA​CAG​AGC​CAC​T
DNAM-1	GTG​GAG​TGG​TTC​AAG​ATC​GGG	GCT​TCC​TTA​TGA​CCA​TGC​CAT
Perforin	ATG​TAA​CCA​GGG​CCA​AAG​TCA	GTG​CCG​TAG​TTG​GAG​ATA​AGC
Granzyme B	GCA​GAT​GCA​GAC​TTT​TCC​TTC	CAC​AGG​GAT​AAA​CTG​CTG​GGT

### 2.5 Flow cytometry

Surface staining was performed by collecting and washing the cells with phosphate-buffered saline (PBS), followed by blocking with 2% bovine serum albumin (BSA) on ice for 30 min. The cells were then incubated with conjugated antibodies on ice for 1 h. For intracellular staining, the cells were fixed and permeabilized using a fixation/permeabilization buffer (BD Biosciences) on ice for 30 min, after which the antibodies were added and incubated under the same conditions. The samples were analyzed by flow cytometry using FACSverse, and the data were analyzed using FlowJo V10 software. For surface staining, APC-conjugated anti-human NKp30 antibody (cat. no. 325210), PE-conjugated anti-human NKp44 antibody (cat. no. 325108), PE-conjugated anti-human NKp46 antibody (cat. no. 331908), PE-conjugated anti-human NKG2D antibody (cat. no. 320806), PE-conjugated anti-human DNAM-1 antibody (cat. no. 338306), and PE-conjugated anti-human 2B4 (cat. no. 329507) were used. For intracellular staining, PE-anti human perforin antibody (cat. no. 353304) and FITC-anti human granzyme B antibody (cat. no. 372206) were used.

For the CD107a degranulation assay, pteryxin-pretreated NK cells were incubated with K562 target cells and FITC-conjugated mouse anti-human CD107a monoclonal antibody (cat. no. 328606) at 37 °C. Then, APC-conjugated mouse anti-human CD56 monoclonal antibody (cat. no. 318310) was added 30 min before detection. The CD107a expression was analyzed using FACSverse. All antibodies used for flow cytometry analyses were purchased from BioLegend.

### 2.6 Western blotting

The cell pellet was lysed in RIPA buffer (100 mM of Tris-HCl (pH 7.4), 50 mM of NaCl, 0.5% NP40, 0.5% sodium deoxycholate, 0.1 mM of Na_3_VO_4_, 50 mM of β-glycerophosphate, and 50 mM of NaF) containing a phosphatase inhibitor cocktail and a protease inhibitor cocktail. The protein concentration was determined using the BCA assay. The proteins were equally loaded onto 10% or 12% SDS polyacrylamide gels and transferred onto polyvinylidene fluoride membranes. After blocking with 5% skimmed milk for 2 h at room temperature, the membranes were incubated overnight with the primary antibodies (1:1000) at 4 °C, followed by washing thrice with TBS-T buffer. Next, the membranes were incubated with appropriate horseradish-peroxidase-conjugated secondary antibodies (1:4000) at room temperature. The immunoreactive protein bands were visualized by enhanced chemiluminescence, and the relative intensities of the bands were quantified using ImageJ.

For Western blotting, we used anti-human-perforin-1 (cat. no. sc-136994) and anti-human-β-actin (cat. no. sc-47778) obtained from Santa Cruz Biotechnology, along with anti-human phospho SAPK/JNK (Thr183/Tyr185) (cat. no. 4668), anti-human SAPK/JNK (cat. no. 9252S), anti-human phospho p44/42 MAPK (ERK1/2) (Thr202/Tyr204) (cat. no. 4370), anti-human p44/42 MAPK (ERK1/2) (cat. no. 4695), anti-human phospho p38 MAPK (Thr180/Tyr182) (cat. no. 4511), anti-human p38 MAPK (cat. no. 8690), anti-human mTOR (cat. no. 2983), phospho-mTOR (Ser2448) (cat. no. 5536), anti-human Vav1 (cat. no. 4657), anti-human phospho Akt (S473) (cat. no. 4060S), anti-human Akt (cat. no. 4691), anti-human MEK1/2 (cat. no. 4694), anti-human phospho MEK1/2 (Ser257/Thr261) (cat. no. 9154), anti-human PI3K (cat. no. 4257), anti-human phospho PI3K (cat. no. 4228), and anti-human granzyme B (cat. no. 4275) purchased from Cell Signaling Technology. Lastly, anti-VAV1 (phospho Y174) (cat. no. ab76225) antibody was purchased from Abcam.

### 2.7 Animals studies

Female Balb/c mice (6 weeks old) were purchased from DBL (South Korea). All mice were housed under standard temperature and humidity conditions at a pathogen-free facility. For the anti-tumor assay, the CT26 cells were injected subcutaneously into the right flanks of the Balb/c mice. On day 18, the mice were euthanized, and the tumor weights and volumes were measured. The tumor sizes were measured using a caliper, and the volume was calculated using the following formula: (major axis) × (minor axis) × height × 0.52. For NK-cell depletion, approximately 100 µL of the anti-asialo GM1 antibody was administered intraperitoneally on the indicated days.

### 2.8 Statistical analysis

All experiments were conducted at least three times and analyzed using GraphPad Prism 9.0 (Dotmatics), and the data were expressed as mean ± standard deviation (SD). Statistical significance between two groups was determined using the unpaired two-tailed Student’s t-test, and multiple-group comparisons were analyzed using one-way ANOVA followed by Dunnett’s post-hoc test. All differences were considered to be statistically significant at *p* < 0.05 (*), *p* < 0.01 (**), *p* < 0.001 (***), or *p* < 0.0001 (****).

## 3 Results

### 3.1 Pteryxin enhances NK-cell-mediated cytotoxicity against tumor cells

**FIGURE 1:**
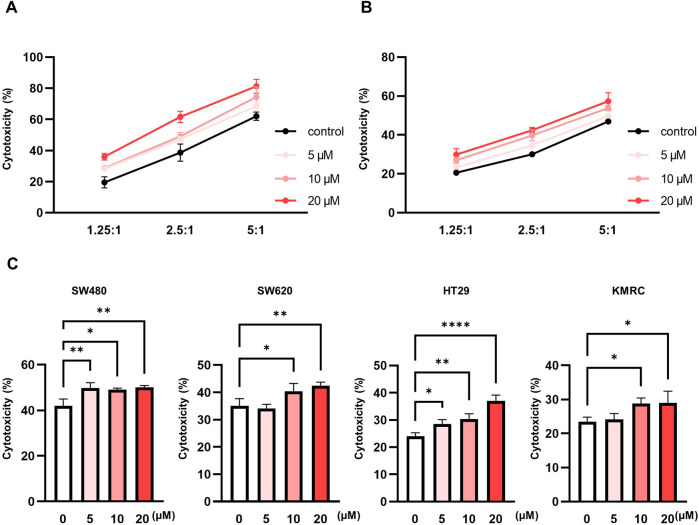
Pteryxin increases the cytotoxic activity of natural killer (NK) cells. Peripheral NK cells and NK-92 cells were exposed to pteryxin at concentrations of 0, 5, 10, and 20 μM. **(A)** Pteryxin-treated peripheral NK cells were cocultured with K562 cells for 2 h, and their cytotoxic activity was analyzed using the calcein-AM release assay. **(B)** Pteryxin-treated NK-92 cells were cocultured with K562 cells for 3 h, and their cytotoxic activity was detected using the calcein-AM release assay. **(C)** Pteryxin-treated NK-92 cells were co-incubated with various colon cancer cell lines for 24 h, and their cytotoxic activities were measured using the lactate dehydrogenase (LDH) release assay. The data are representative of three independent experiments (n = 3 biological replicates) and are presented as mean ± standard deviation (SD). Statistical significance was determined using one-way ANOVA followed by Dunnett’s post-hoc test.

To investigate the immunomodulatory potential of pteryxin on peripheral NK (pNK) cells, we first determined the optimal treatment concentration. Here, the pNK cells were exposed to increasing concentrations of pteryxin (up to 160 μM) for 24 h and 48 h, followed by viability assessment using the CCK-8 assay. A marked reduction in cell viability was observed at concentrations ≥40 μM at both time points (Supplementary Figure S1A), which allowed establishing a maximum concentration of 20 μM for the subsequent analyses. To evaluate the NK-cell cytotoxic functions, a calcein-AM release assay was employed. Here, the pNK cells were treated with 0, 5, 10, or 20 μM of pteryxin for 48 h and subsequently cocultured with K562 target cells for 2 h at effector-to-target (E:T) ratios of 1.25:1, 2.5:1, and 5:1. The pteryxin treatment resulted in dose-dependent enhancement of the cytotoxic activity across all tested E:T ratios (Figure 1A). Additionally, similar experiments were conducted using the NK-92 cell line, where the optimal concentration of pteryxin was established via the CCK-8 assay following 24 h and 48 h treatments (Supplementary Figure S1B). The NK-92 cells pretreated with pteryxin were cocultured with K562 cells at varying E:T ratios, and the cytotoxic activity was quantified using the calcein-AM assay. The pteryxin-treated NK-92 cells exhibited enhanced cytotoxicity against the K562 cells (Figure 1B). Furthermore, to assess the potential of pteryxin in augmenting NK-cell-mediated cytotoxicity against solid tumors, the pteryxin-treated NK-92 cells were cocultured with multiple colorectal cancer cell lines, including SW480, SW620, HT-29, and KMRC, at a fixed E:T ratio of 5:1. Here, pteryxin was observed to significantly enhance NK-92-mediated cytotoxic responses against all tested tumor cell lines (Figure 1C). Collectively, these results demonstrate that pteryxin enhances NK cell effector function in a dose-dependent manner and exerts broad-spectrum cytotoxic effects against both hematologic and solid tumor targets.

### 3.2 Pteryxin induces expression of activating receptors in NK cells

**FIGURE 2:**
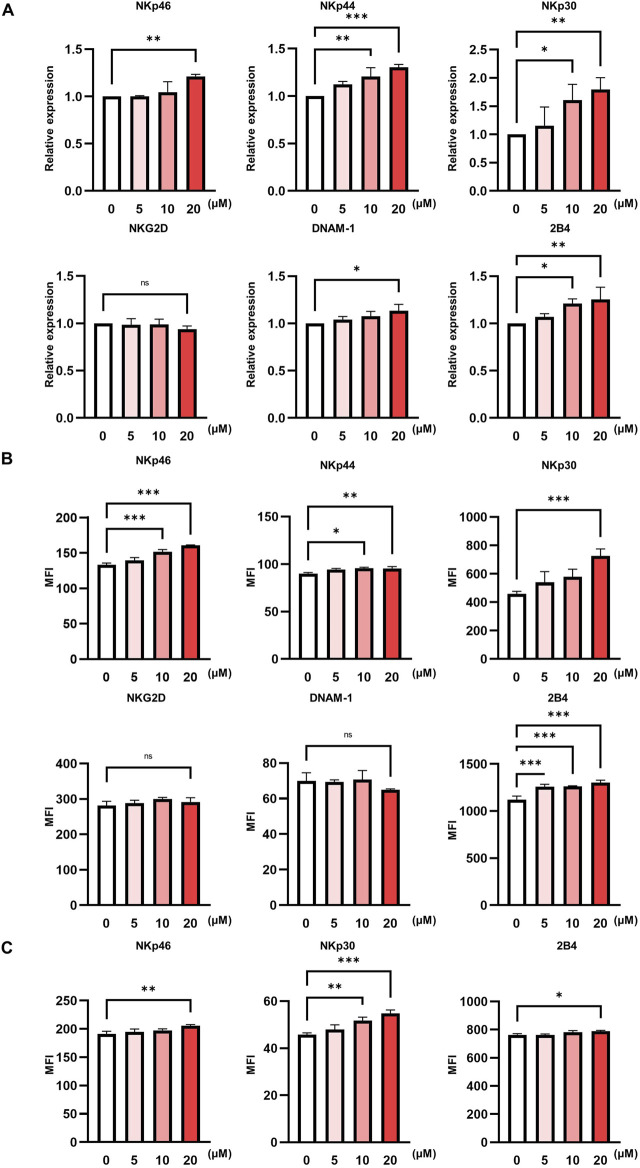
Pteryxin enhances the activating receptors of NK cells. NK-92 cells were exposed to pteryxin at concentrations of 0, 5, 10, and 20 μM for 48 h, and the major NK activating receptors were analyzed. **(A)** The mRNA expression levels of NKp46, NKp44, NKp30, NKG2D, DNAM-1, and 2B4 were measured by quantitative real-time polymerase chain reaction (qRT-PCR). **(B)** The protein levels of these receptors were measured by FACS analysis. **(C)** The protein levels of the activating receptors NKp46, NKp30, and 2B4 were evaluated in primary NK cells. The data represent the fold changes of expression and mean fluorescence intensities of the receptors. The data are representative of three independent experiments (n = 3 biological replicates) and are presented as mean ± SD. Statistical significance was determined using one-way ANOVA followed by Dunnett’s post-hoc test.

Since the cytotoxic functions of NK cells are critically regulated by the dynamic balance between the activating and inhibitory receptors (Chan et al., 2014), we next assessed whether pteryxin could modulate the expression of key NK-cell-activating receptors. Quantitative RT-PCR analysis revealed that the mRNA expression levels of NKp46, NKp44, NKp30, NKG2D, DNAM-1, and 2B4 were upregulated in a dose-dependent manner following pteryxin treatment (Figure 2A). Consistently, the flow cytometry analysis demonstrated a marked increase in NKp30 receptor expression and slight increases in NKp44, NKp46, and 2B4 receptor expression in a dose-dependent manner (Figure 2B). Moreover, pteryxin also induced NKp30, NKp46, and 2B4 receptor expression in the pNK cells in a dose-dependent manner (Figure 2C). These findings suggest that pteryxin potentially upregulates the activating receptors to promote NK-cell activation.

### 3.3 Pteryxin promotes lytic granule expression and degranulation in NK-92 cells

**FIGURE 3:**
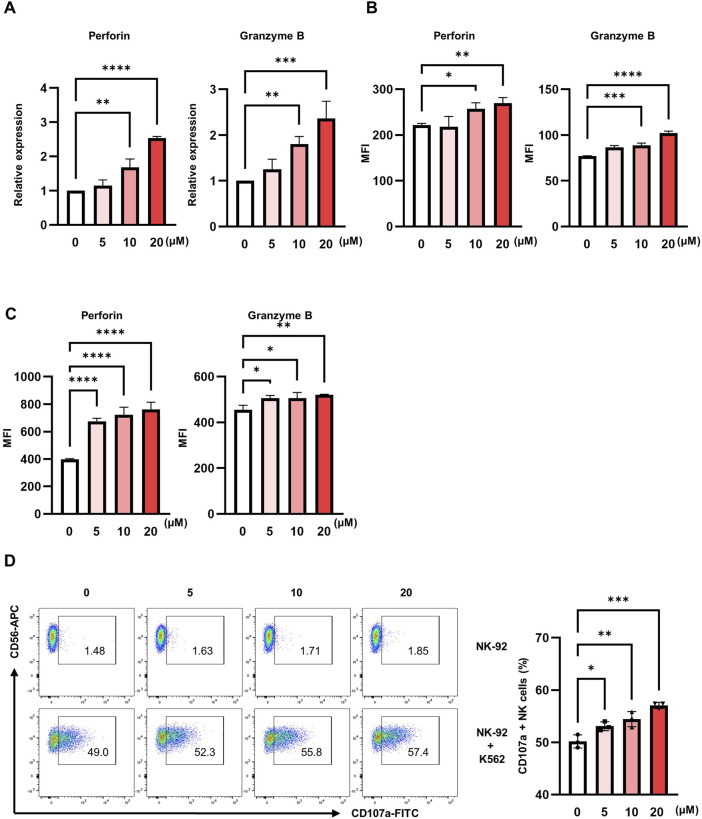
Pteryxin regulates the cytolytic granules of NK cells. NK-92 cells were exposed to pteryxin at concentrations of 0, 5, 10, and 20 μM for 48 h. **(A)** The mRNA expression levels of these cells were detected by qRT-PCR, and **(B)** the protein expression levels of perforin and granzyme B were obtained by FACS analysis. **(C)** The protein levels of perforin and granzyme B were also evaluated in the primary NK cells. The data represent the fold changes of expression and mean fluorescence intensities of the cells. **(D)** Pteryxin-treated NK-92 cells were cocultured with K562 cells for 3 h at a ratio of 1:1. The CD107a^+^ and CD56^+^ cells were detected by FACS analysis. The left panel shows the dot plot of a representative image, while the right panel presents the average percentage of CD107a^+^ NK cells. The data are representative of three independent experiments (n = 3 biological replicates) and are presented as mean ± SD. Statistical significance was determined using one-way ANOVA followed by Dunnett’s post-hoc test.

The NK cells primarily exert cytotoxic effects through the exocytosis of lytic granules containing effector molecules, such as perforin and granzymes (Krzewski and Coligan, 2012). To evaluate whether pteryxin modulates the expression of these cytotoxic mediators, NK-92 cells were treated with increasing concentrations of pteryxin (0, 5, 10, and 20 μM) for 48 h. Subsequently, the mRNA and protein expression levels of perforin and granzyme B were analyzed by quantitative RT-PCR and flow cytometry, respectively. Both perforin and granzyme B mRNA levels were significantly upregulated in a dose-dependent manner along with substantial increases in their corresponding protein expression (Figures 3A,B). Similar patterns of upregulation were observed in the pNK cells following pteryxin treatment (Figure 3C). To further assess degranulation activity, CD107a surface expression was measured as a marker of granule exocytosis; here, the NK-92 cells were cocultured with K562 target cells at an E:T ratio of 1:1 for 3 h, and the CD107a expression on the CD56^+^ cells was analyzed by flow cytometry. A modest increase in the frequency of the CD107a^+^CD56^+^ cells was observed in response to pteryxin treatment (Figure 3D). These results suggest that pteryxin enhances the cytotoxic activity of NK cells by inducing the expression and production of lytic granules through exocytosis.

### 3.4 Pteryxin modulates NK-cell cytotoxicity through the ERK/AKT signaling pathways

**FIGURE 4:**
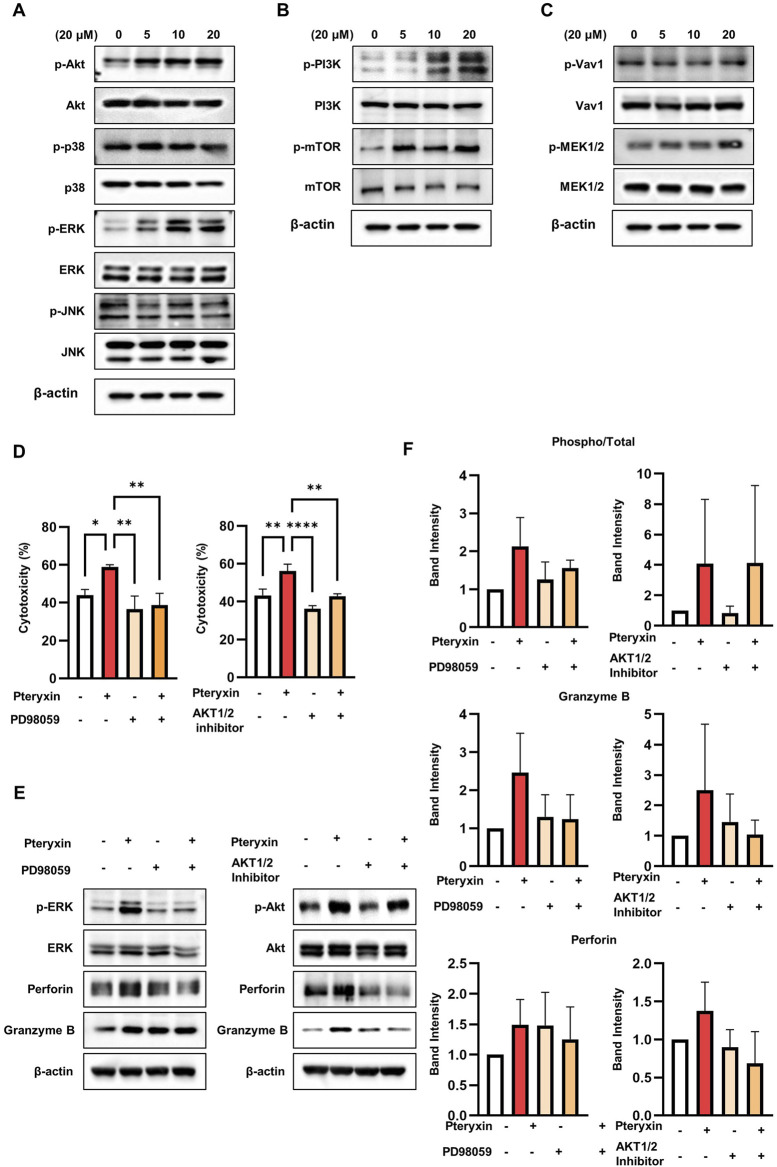
Pteryxin effects on the ERK/AKT signaling pathways. NK-92 cells were exposed to pteryxin at concentrations of 0, 5, 10, and 20 μM for 48 h. **(A)** The phosphorylation levels of AKT, p38, ERK, and JNK were analyzed using Western blotting, followed by **(B)** detection of the phosphorylation levels of mTOR and PI3K pathways upstream of the AKT pathway. **(C)** The phosphorylation levels of vav-1 and MEK1/2 upstream of the ERK pathway were analyzed. **(D)** NK-92 cells were treated with or without 20 μM of pteryxin followed by PD98059 or AKT1/2 inhibitors before being cocultured with K562 cells for 3 h at a ratio of 5:1. The cytotoxic activity was assessed using the calcein-AM release assay. **(E)** The total and phosphorylated levels of ERK and AKT along with those of perforin and granzyme B were analyzed using Western blotting. **(F)** The Western blot band intensities were normalized to those of actin as the reference. The data represent three independent experiments (n = 3 biological replicates) and are presented as mean ± SD. Statistical significance was determined using one-way ANOVA followed by Dunnett’s post-hoc test.

Previous studies have established the MAPK pathway as a critical regulator of NK-cell-mediated cytotoxicity (Chen et al., 2007; Colucci et al., 2002). To determine whether pteryxin modulates MAPK signaling in the NK cells, we treated NK-92 cells with increasing concentrations of pteryxin (0, 5, 10, and 20 μM) for 48 h, followed by immunoblotting analysis of the key signaling proteins. Pteryxin treatment induced dose-dependent increases in the phosphorylation of ERK and AKT, whereas the phosphorylation levels of p38 and JNK remained largely unchanged (Figure 4A). In addition, pteryxin increased ERK and AKT phosphorylation in primary NK cells (Supplementary Figure S2A). Moreover, pteryxin promoted the phosphorylation of mTOR and PI3K as upstream regulators of AKT (Figure 4B) as well as MEK1/2 as an upstream mediator of ERK (Figure 4C). To further investigate whether the enhancement of NK-cell cytotoxicity by pteryxin was mediated via the ERK and/or AKT signaling pathways, we employed pharmacological inhibitors specific to each pathway. The NK-92 cells were pretreated with 10 μM of PD98059 (an ERK inhibitor) or AKT1/2 inhibitor prior to pteryxin administration. Notably, both inhibitors significantly attenuated the pteryxin-induced cytotoxic activity of the NK-92 cells (Figure 4D). Moreover, perforin expression levels were reduced by the ERK/AKT inhibitors (Figure 4E). These results suggest that pteryxin enhances the cytotoxic activity of the NK cells by inducing perforin through the ERK/AKT signaling pathways.

### 3.5 Pteryxin reduces tumor growth by augmenting NK-cell-mediated cytotoxicity in mice

**FIGURE 5:**
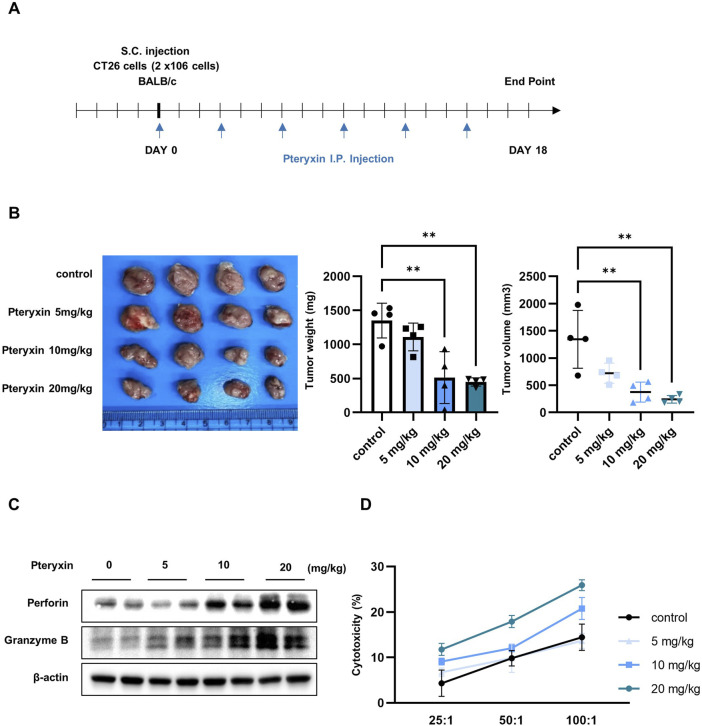
Pteryxin inhibits tumor growth in the CT26 mouse model. **(A)** Experimental scheme for determining the optimal pteryxin dosage. The CT26 cells were subcutaneously injected into Balb/c mice on day 0 followed by intraperitoneal injections of pteryxin every 3 days. On day 18, the mice were euthanized, and the tumor volumes and weights were measured. **(B)** Representative tumor images are shown in the left panel, while the average tumor weight and volume are presented in the bar graphs on the right side. **(C)** Expression levels of perforin and granzyme B were analyzed by Western blotting. **(D)** Splenocytes and YAC-1 cells were additionally cocultured for 24 h, and their cytotoxic activities were analyzed using the LDH assay. The data represent three independent experiments (n = 4 biological replicates) and are presented as mean ± SD. Statistical significance was determined using one-way ANOVA followed by Dunnett’s post-hoc test.

To evaluate the potential of pteryxin to suppress tumor growth in a mouse model, CT26 cells were injected subcutaneously into mice and pteryxin was administered intraperitoneally every 3 days. The mice were randomly assigned into four groups receiving 0, 5, 10, or 20 mg/kg of pteryxin injection. On day 18, the mice were euthanized, and the tumor volumes and weights were measured (Figure 5A). Both tumor weight and volume showed significant reductions in a dose-dependent manner (Figure 5B). To investigate whether the NK cells were involved in tumor growth inhibition, splenocytes were isolated from the spleens of the mice. The expression levels of perforin and granzyme B were higher in the splenocytes of pteryxin-administered mice (Figure 5C). Furthermore, when the splenocytes were cocultured with YAC-1, a mouse leukemia cell line, the cytotoxicity was enhanced in the splenocytes from pteryxin-administered mice compared to the control group (Figure 5D).

**FIGURE 6:**
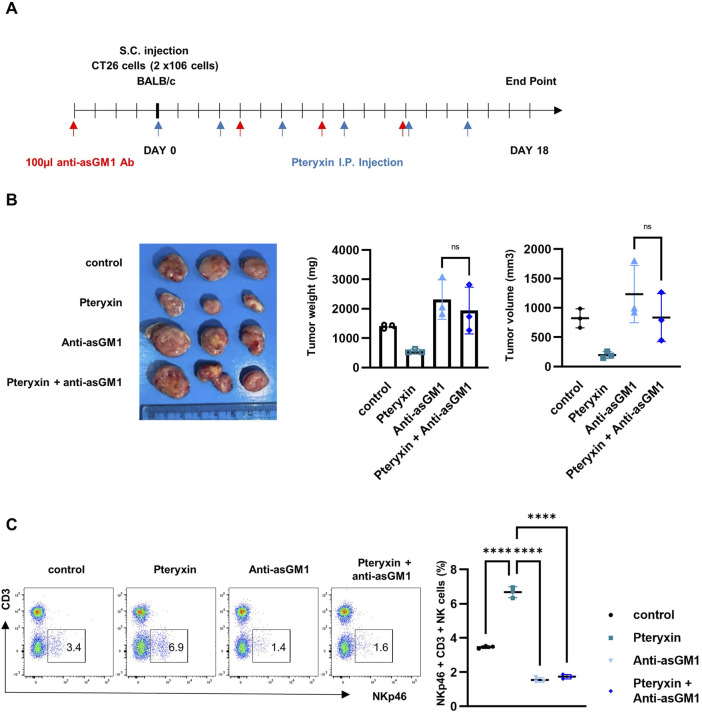
Anticancer effects of pteryxin in mice are eliminated by NK-cell depletion. **(A)** The experimental scheme shows that CT26 cells were injected on day 0 and that pteryxin was administered every 3 days; to deplete the NK cells, 100 µL of anti-asGM1 antibody was injected on days −4, 4, 8, and 12. **(B)** The tumor images are shown in the left panel, while the average tumor weight and volume are presented in the bar graphs on the right side. **(C)** Splenocytes were isolated from each mouse, and the NK-cell population was analyzed using FACS. The left panel shows representative images from FACS, while the right panel shows the average NK-cell population. The data represent three independent experiments (n = 3 biological replicates) and are presented as mean ± SD. Statistical significance was determined using one-way ANOVA followed by Dunnett’s post-hoc test.

To further determine whether the anticancer effects of pteryxin were mediated by NK cells, we used an anti-asialo GM1 (asGM1) antibody to deplete the NK cells. Here, the CT26 injected mice were administered pteryxin and/or an anti-asGM1 antibody (Figure 6A). Significant reductions in the tumor volume and weight were observed in the group treated with pteryxin relative to the controls. However, the anticancer effects of pteryxin were completely abrogated in mice depleted of NK cells using the anti-asGM1 antibody (Figure 6B). FACS analysis showed a slight increase in the frequency of CD3-NKp46^+^ NK cells in the spleens of pteryxin-treated mice. In contrast, this NK cell population was completely abolished in mice coadministered pteryxin and anti-asGM1 antibody (Figure 6C). Collectively, these findings indicate that pteryxin suppresses tumor growth by enhancing the cytotoxic potential of the NK cells.

## 4 Discussion

In this study, we discovered that pteryxin enhances the cytotoxicity of NK cells by modulating the ERK/AKT signaling pathway. Pteryxin is a natural compound in the coumarin group that exhibits a broad spectrum of bioactivities, including antioxidant, anti-inflammatory, anti-Alzheimer’s, antihyperglycemic, antiseizure, and antiuropathogenic effects (Patel and Patel, 2024; Orhan et al., 2017). Although pteryxin shows considerable medicinal potential, its antitumor effects have not been studied previously. In the present study, pteryxin was found to enhance the cytotoxic abilities of both NK-92 and primary human NK cells, leading to more efficient killing of K562 leukemia cells. Additionally, pteryxin increased the cytotoxicity of the NK-92 cells against several colon cancer cell lines. These results suggest that pteryxin may serve as a valuable agent for enhancing NK-cell-mediated cytotoxicity, potentially offering a therapeutic strategy for treating various types of cancer. Furthermore, *in vivo* studies using a CT26-bearing mouse model demonstrated that pteryxin effectively inhibited tumor growth in a dose-dependent manner. At a dose of 20 mg/kg, there were no observable signs of toxicity. Additionally, the anticancer effects of pteryxin were completely reversed in mice that underwent NK-cell depletion with anti-asGM1 antibody, further supporting the notion that pteryxin enhances NK-cell-mediated tumor surveillance.

In this study, we demonstrated that pteryxin enhances the cytotoxic activity of NK cells by upregulating the expression of several activating receptors, including NKp46, NKp44, NKp30, DNAM-1, and 2B4. These receptors are known to recognize ligands on the target cells, leading to NK-cell activation (Barrow et al., 2019). Importantly, pteryxin significantly increased the levels of these receptors, suggesting that pteryxin modulates NK-cell activation by promoting the expression of receptors crucial for cytotoxicity. The degranulation of NK cells is a major mechanism underlying their cytotoxic functions (Voskoboinik et al., 2015; Wong and Ding, 2023). Accordingly, our study revealed that pteryxin increased the expression of CD107a as well as upregulated the expression of perforin and granzyme B in NK-92 and primary NK cells; this indicates that pteryxin enhances the activating receptor expression and promotes NK-cell degranulation, further contributing to their cytotoxic potential.

The downstream effectors of these activating receptors, such as MAPK and AKT, are crucial for promoting the cytotoxic functions of NK cells (Chen et al., 2007). Accordingly, we observed that pteryxin specifically regulates the AKT and ERK signaling pathways without significantly impacting the p38 and JNK pathways. Inhibition of either the ERK or AKT pathway completely abolished the pteryxin-induced enhancement of NK-cell cytotoxicity and expression of perforin. These findings suggest that these pathways are at least partly associated with the enhancement of NK-cell cytotoxicity induced by pteryxin.

In summary, our results indicate that pteryxin activates NK cells in NK cell lines, primary NK cells, and in mice. Pteryxin treatment increased the expression of NK activating receptors, such as NKp30, NKp46, and 2B4, through the ERK/AKT signaling pathway, leading to elevated expression of perforin and granzyme B. These findings reveal a previously unrecognized immunomodulatory role of pteryxin and support its potential as a novel agent for enhancing NK-cell-based antitumor immunity.

Aside from the significant findings here, this study has some limitations. Given that there are numerous other inhibitory and activating receptors beyond those examined herein, further research efforts are needed to confirm their expression and to identify additional signaling pathways influenced by pteryxin. Future studies should therefore investigate the effects of pteryxin for a broader range of cancer cell lines and other aspects of NK-cell cytotoxicity, such as cytokine profiles and death receptor pathways, to gain a more comprehensive understanding of the immunomodulatory effects. While our *in vivo* results indicate that pteryxin enhances NK-cell-mediated antitumor responses, systemic administration may also influence other immune populations; furthermore, anti-asGM1-mediated NK depletion can affect the basophils and subsets of innate lymphoid cells, limiting the absolute NK specificity of pteryxin. To address these concerns, future studies employing more specific NK depletion strategies or NOD mice will be important to delineate the precise NK-cell-intrinsic mechanisms of pteryxin. Moreover, given the broad pharmacological properties of pteryxin (Patel and Patel, 2024), future studies should further assess the pharmacokinetic characteristics and explore its potential as an adjuvant in combination with immune checkpoint inhibitors or CAR-NK cell therapies to enhance clinical applicability.

## Data Availability

The original contributions presented in the study are included in the article/Supplementary Material, and any further inquiries can be directed to the corresponding authors.
