# Supplementary material for: Pteryxin enhances human NK-cell cytotoxicity by upregulating NKp30, NKp46, and 2B4 via ERK/AKT signaling
Source: Front Pharmacol. 2026 Jan 21;16:1698826. doi: 10.3389/fphar.2025.1698826 (PMC12868148; doi:10.3389/fphar.2025.1698826)
Supplement: Supplementary file 1 [file Table1.docx]

Supplementary Material

**Supplementary Table 1. Reagent & Antibody list**

| **Name** | **CATALOG NUMBER** | **COMPANY** | **CLONE** |
| --- | --- | --- | --- |
| FBS | A5256701 | gibco |  |
| Horse serum | 26050088 | gibco |  |
| myo-inositol | [I5125](https://www.sigmaaldrich.com/KR/ko/product/sigma/i5125) | sigma-aldrich |  |
| mercaptoethanol | [M3148](https://www.sigmaaldrich.com/KR/ko/product/sigma/m3148) | sigma-aldrich |  |
| folic acid | [F8758](https://www.sigmaaldrich.com/KR/ko/product/sigma/f8758) | sigma-aldrich |  |
| immunocult NK cell expansion kit | 100-0711 | Stemcell technologies |  |
| IL-2 | 200-02 | peprotech |  |
| CCK-8 reagent | CK04 | Dojindo molecular technologies |  |
| CytoTox 96 Non-Radiative cytotoxicity Assay | G1780 | Promega |  |
| Calcein-AM | C34852 | sigma-aldrich |  |
| RNA extraction kit | RP101-100 | Biofact |  |
| p44/42 MAPK (Erk1/2) (137F5) Rabbit mAb | 4695 | CST | 137F5 |
| Phospho-p44/42 MAPK (Erk1/2) (Thr202/Tyr204) (D13.14.4E) XP® Rabbit mAb | 4370 | CST | D13.14.4E |
| p38 MAPK (D13E1) XP® Rabbit mAb | 8690 | CST | D13E1 |
| Phospho-p38 MAPK (Thr180/Tyr182) (D3F9) XP® Rabbit mAb | 4511 | CST | D3F9 |
| SAPK/JNK Antibody | 9252 | CST |  |
| Phospho-SAPK/JNK (Thr183/Tyr185) (81E11) Rabbit mAb | 4668 | CST | 81E11 |
| Akt (pan) (C67E7) Rabbit mAb | 4691 | CST | C67E7 |
| Phospho-Akt (Ser473) (D9E) XP® Rabbit mAb | 4060 | CST | D9E |
| β-actin | sc-47778 | Santa Cruz | C4 |
| mTOR (7C10) Rabbit mAb | 2983 | CST | 7C10 |
| Phospho-mTOR (Ser2448) (D9C2) XP® Rabbit mAb | 5536 | CST | D9C2 |
| MEK1/2 (L38C12) Mouse mAb | 4694 | CST | L38C12 |
| Phospho-MEK1/2 (Ser217/221) (41G9) Rabbit mAb | 9154 | CST | 41G9 |
| Vav1 (D45G3) Rabbit mAb | 4657 | CST | D45G3 |
| Anti-VAV1 (phospho Y174) antibody [EP510Y] | ab76225 | abcam | EP510Y |
| anti-perforin 1 antibody | sc-136994 | Santa Cruz | F-1 |
| anti- Granzyme B antibody | 4275S | CST |  |
| APC anti-human CD337 (NKp30) | 325210 | biolegend | P30-15 |
| PE anti-human CD336 (NKp44) | 325108 | biolegend | P44-8 |
| PE anti-human CD335 (NKp46) | 331908 | biolegend | 9E2 |
| PE anti-human CD314 (NKG2D) | 320806 | biolegend | 1D11 |
| PE anti-human perforin antibody | 353304 | biolegend | B-D48 |
| APC anti-human CD56 | 318310 | biolegend | HCD56 |
| FITC anti-human CD107a | 328606 | biolegend | H4A3 |
| FITC anti-human/mouse Granzyme B recombinant antibody | 372204 | biolegned | QA16A02 |
| APC-anti-mouse CD3 antibody | 349201 | BD | SK7 |
| [BD Pharmingen™ PE Rat Anti-Mouse CD335 (NKp46)](https://www.bdbiosciences.com/ko-kr/products/reagents/flow-cytometry-reagents/research-reagents/single-color-antibodies-ruo/pe-rat-anti-mouse-cd335-nkp46.560757) | 560757 | BD | 29A1.4 |
