# Supplementary material for: Pteryxin enhances human NK-cell cytotoxicity by upregulating NKp30, NKp46, and 2B4 via ERK/AKT signaling
Source: Front Pharmacol. 2026 Jan 21;16:1698826. doi: 10.3389/fphar.2025.1698826 (PMC12868148; doi:10.3389/fphar.2025.1698826)
Supplement: Supplementary file 2 [file Presentation1.pptx]

## Slide 1
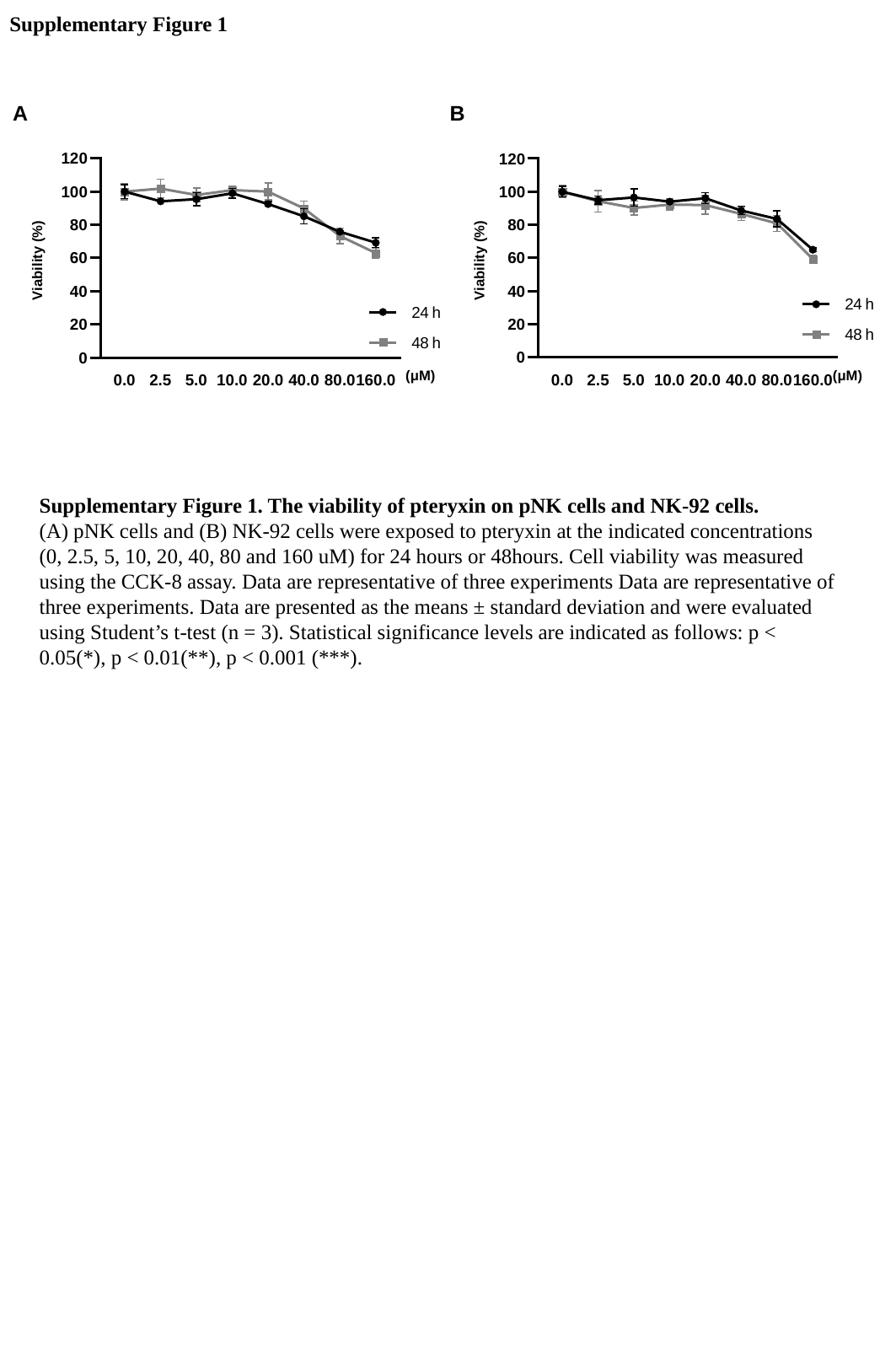

Supplementary Figure 1
A
B
Viability (%)
Viability (%)
(μM)
(μM)
Supplementary Figure 1. The viability of pteryxin on pNK cells and NK-92 cells.
(A) pNK cells and (B) NK-92 cells were exposed to pteryxin at the indicated concentrations (0, 2.5, 5, 10, 20, 40, 80 and 160 uM) for 24 hours or 48hours. Cell viability was measured using the CCK-8 assay. Data are representative of three experiments Data are representative of three experiments. Data are presented as the means ± standard deviation and were evaluated using Student’s t-test (n = 3). Statistical significance levels are indicated as follows: p < 0.05(*), p < 0.01(**), p < 0.001 (***).

## Slide 2
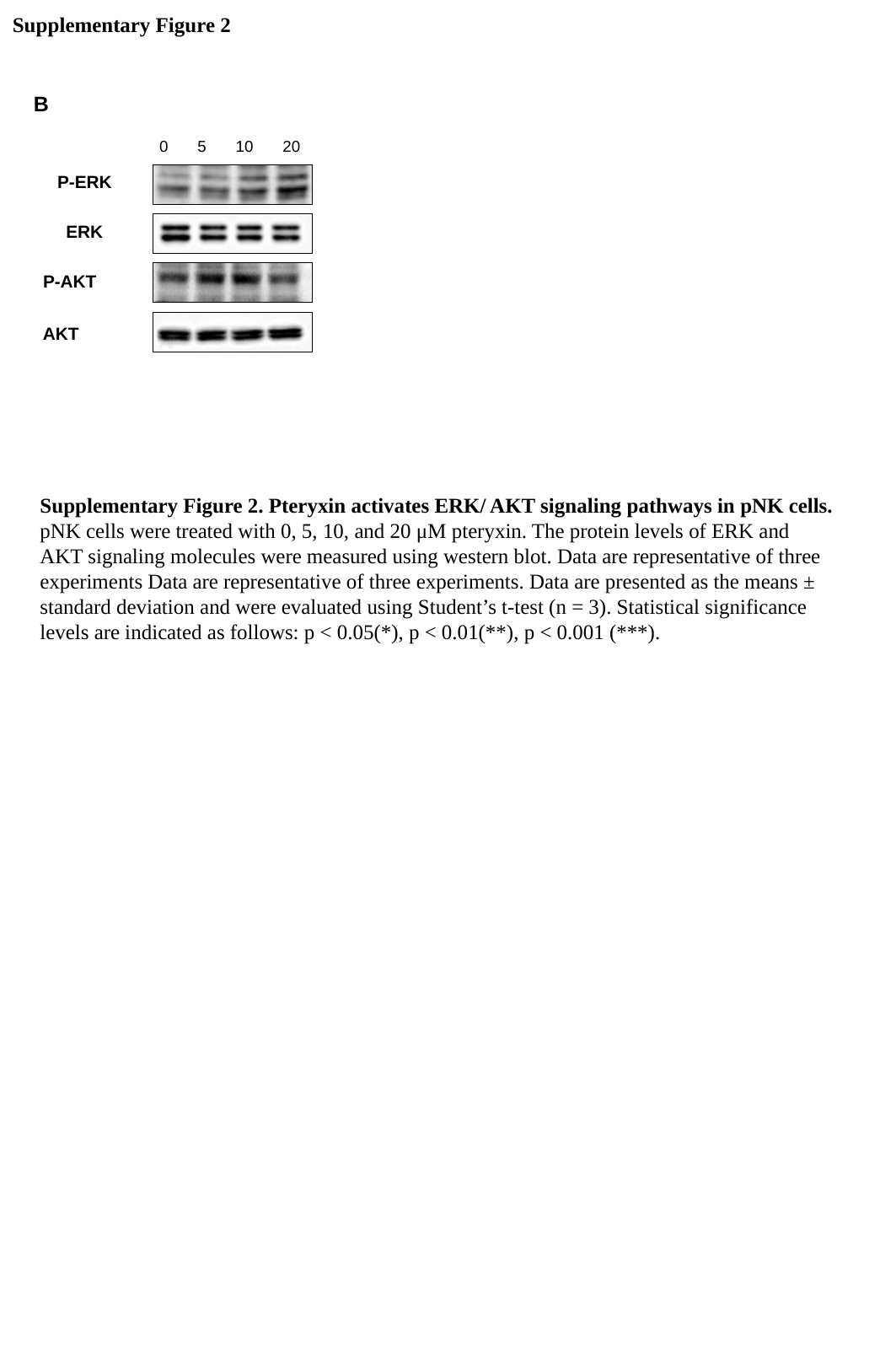

Supplementary Figure 2
B
0
5
10
20
P-ERK
ERK
P-AKT
AKT
Supplementary Figure 2. Pteryxin activates ERK/ AKT signaling pathways in pNK cells. pNK cells were treated with 0, 5, 10, and 20 μM pteryxin. The protein levels of ERK and AKT signaling molecules were measured using western blot. Data are representative of three experiments Data are representative of three experiments. Data are presented as the means ± standard deviation and were evaluated using Student’s t-test (n = 3). Statistical significance levels are indicated as follows: p < 0.05(*), p < 0.01(**), p < 0.001 (***).

## Slide 3
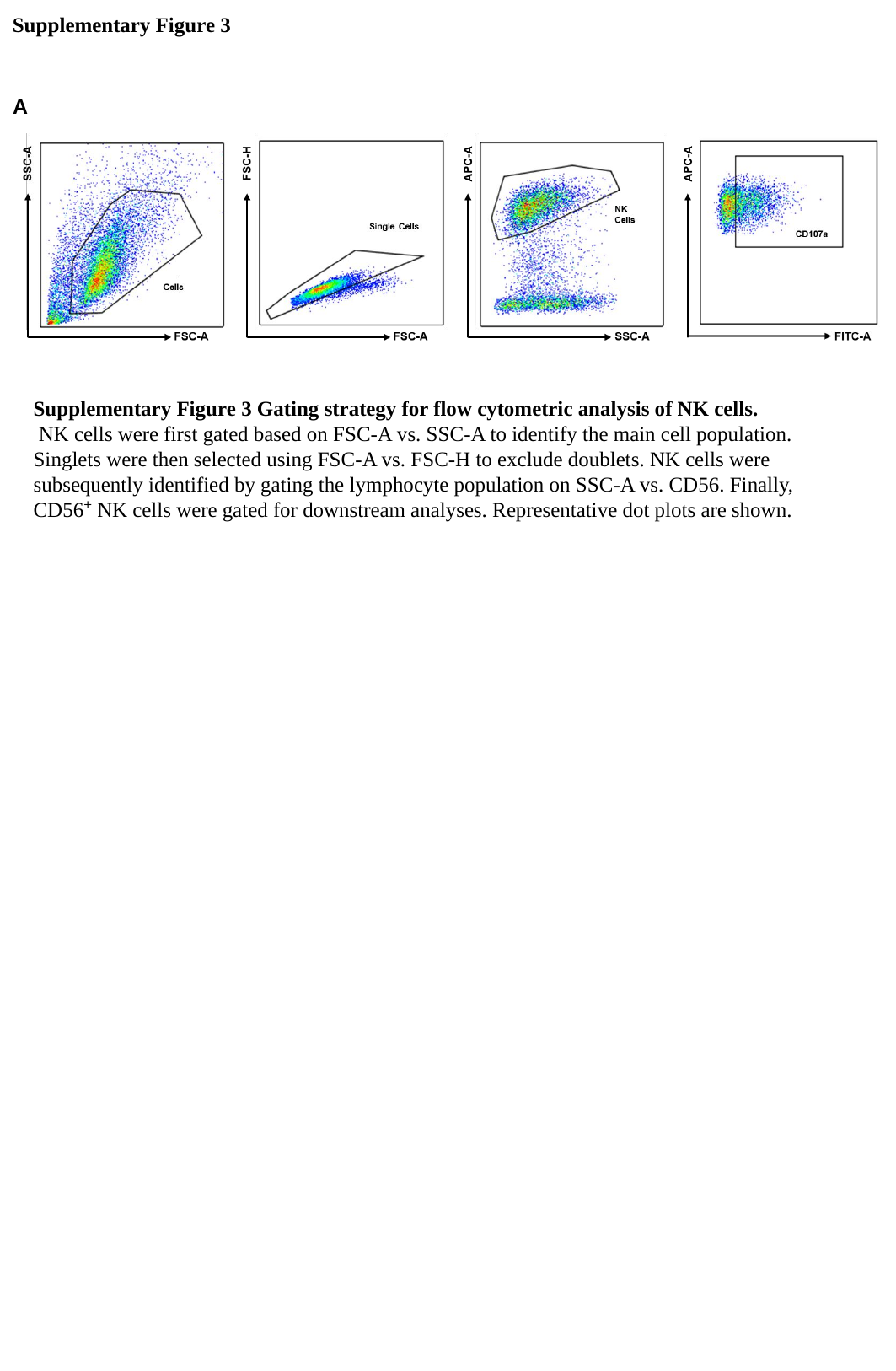

Supplementary Figure 3
A
Supplementary Figure 3 Gating strategy for flow cytometric analysis of NK cells.
 NK cells were first gated based on FSC-A vs. SSC-A to identify the main cell population. Singlets were then selected using FSC-A vs. FSC-H to exclude doublets. NK cells were subsequently identified by gating the lymphocyte population on SSC-A vs. CD56. Finally, CD56⁺ NK cells were gated for downstream analyses. Representative dot plots are shown.
